# Student Perceptions of 360^o^ (3D) Imaged‐Models Used for Fixed Prosthodontics Simulation Education: Enhancing Visualisation, Understanding and Self‐Assessment

**DOI:** 10.1111/eje.70128

**Published:** 2026-03-06

**Authors:** Manal Ablal, Barry Quinn, Ethan Hearty, Michael Aspden

**Affiliations:** ^1^ The School of Dentistry, Institute of Life Course and Medical Sciences The University of Liverpool Pembroke Place Liverpool UK

**Keywords:** 3D imaged models, dental education, self‐assessment, simulation learning, understanding, visualisation

## Abstract

**Introduction:**

This study evaluated and compared the effectiveness of a novel 3D imaged models (3D IMs) and physical reference models in supporting student learning of indirect tooth preparations.

**Materials and Methods:**

Fourth‐year dental students from 2021 (*N* = 69) and 2022 (*N* = 54) received access to 3D IMs and real‐sized plastic models featuring inlays, veneers and other restorations. A 5‐point Likert scale questionnaire assessed the perceived impact of both resources on students' learning. Open‐ended responses were thematically analysed to identify common themes in student perceptions.

**Results:**

Mann–Whitney *U* test indicated clear differences between the two cohorts. The 2022 students rated the 3D IMs more positively for visualisation, understanding and self‐assessment skills (*p* < 0.001). While both cohorts agreed that physical models aided hands‐on learning, the 2022 cohort gave consistently higher ratings to 3D IMs. Spearman's rank correlations showed meaningful associations between Likert items. Thematic analysis showed mixed perceptions and access challenges in 2021, along with suggestions for improving physical models. In contrast, the 2022 cohort expressed more positive views and recommended enhancements such as annotations, measurement indicators and broader integration of 3D IMs into the curriculum.

**Conclusion:**

This study suggests that 3D IMs can support students' visualisation, understanding and self‐assessment of tooth preparations, while physical models remain valuable for hands‐on learners. Adapting teaching tools to accommodate diverse learning preferences and expanding access across the curriculum may further enhance learning outcomes. Further research with larger cohorts and longitudinal follow‐up is needed to confirm these preliminary findings and assess their long‐term educational impact.

## 1 Introduction

In the wake of the global pandemic and the rapid transition to online learning, the field of education has undergone a transformative shift [1, 2, 3, 4]. This shift has challenged educators to explore innovative approaches for pedagogical enhancement, particularly in fields requiring hands‐on training, such as dentistry. Within dental education, the communication of complex anatomical and procedural concepts has long posed a challenge, necessitating the adoption of innovative teaching methodologies. Among these, the integration of digital technologies, such as 3D scanning and printing, has emerged as a promising avenue for enhancing student learning [5, 6, 7, 8].

While previous research in dental education has explored the use of 3D‐printed models to aid in teaching fixed prosthodontics [9, 10, 11], these approaches focused on physical replicas rather than interactive digital models. In contrast, this study developed and evaluated the use of novel 3D imaged models (3D IMs); virtual, annotated and fully rotatable resources specifically designed to enhance student understanding, visualisation and self‐assessment of indirect tooth preparations. To our knowledge, few studies have systematically investigated the educational impact of interactive 3D IMs within pre‐clinical fixed prosthodontics training.

One of the fundamental challenges encountered by dental students revolved around the creation of tooth preparations (TPs) that adhere to some gold standard criteria [12]. This often led students to cope with self‐assessment as they struggled to identify subtle errors that could compromise the longevity and fit of indirect restorations [13]. Traditional teaching methods often rely on physical models and faculty feedback, but these approaches may not fully address the diverse learning needs of students. As a result, many students struggle with self‐assessment and identifying subtle errors in their TPs, which can compromise their overall quality [14].

This study aimed to address the gap in understanding the potential of novel 3D imaged models (3D IMs) in pre‐clinical fixed prosthodontics (FPs) teaching and learning. By evaluating student perceptions on 3D IMs, as compared to their experiences with physical models, we explored how these digital models could enhance understanding of tooth preparations and visualisation of specific TP details. Drawing inspiration from observations made during the delivery of the fixed prosthodontics course, where students often struggled with visualising and comprehending TPs, these novel 3D IMs provided valuable insights into the benefits and limitations of integrating digital technologies in FPs, ultimately highlighting the need for more effective teaching resources like 3D IMs. Among their key advantages, 3D IMs can be freely rotated 360 degrees, zoomed in and out and feature clearly highlighted components of the TPs, allowing easy differentiation between prepared and unprepared surfaces. These models are also annotated to explain the different structural elements and the required amount of tooth reduction, adding further educational value. An additional benefit is their accessibility; students can interact with them anytime and anywhere via mobile devices, promoting flexible, self‐directed learning.

### 1.1 Research Question

Do 3D imaged models improve students' perceived visualisation, understanding and self‐assessment of tooth preparations compared with physical models in pre‐clinical fixed prosthodontics training?

### 1.2 Null Hypothesis

There's no difference in students' perceived visualisation, understanding and self‐assessment of tooth preparations compared with physical models in pre‐clinical fixed prosthodontics training.

## 2 Materials and Methods

### 2.1 Study Design

This study used a within‐subjects design to assess dental students' perceptions of 3D IMs compared to physical models in a pre‐clinical FPs course. Both were provided simultaneously, allowing direct comparison of student perceptions within the same learning context. This concurrent approach minimised the potential bias of learning effects associated with sequential exposure. Conducted over two academic years (2021 and 2022) with fourth‐year dental students (*N* = 123), the study primarily focused on student perceptions while also documenting course outcomes to contextualise the findings.

### 2.2 Ethical Consideration

Ethical approval was granted by the related Ethics Committee (study ID: 10644). Data were collected anonymously to ensure that no participant could be identified. Informed consent was obtained, and participants were provided with Participant Information prior to commencing the study.

### 2.3 Study Participants

Participants were 4th‐year dental students enrolled in the bridge course during the 2021 (*N* = 69) and 2022 (*N* = 54) academic years. This pre‐clinical course focuses on teaching students how to prepare teeth for various indirect restoration designs in a simulated setting.

The 2021 cohort started their course following the end of the pandemic in March 2021. These students transitioned back to face‐to‐face teaching and learning, whereas the 2022 cohort entered the program in a post‐pandemic environment.

### 2.4 Selection of Tooth Preparation Designs

Four tooth preparation designs were selected for the study: anterior ceramic veneer, ceramic/composite inlays, ceramic onlay and anterior minimal preparation bridge preparations. These were identified based on students' performance during the practical sessions in the simulation suite. During those sessions, student preparations were assessed by tutors and scored using Liftupp, an online assessment and student development tool. The designs most frequently associated with lower performance scores and greater student difficulty were chosen for inclusion, as they represented areas where additional support was most needed.

### 2.5 Preparation of 3D Imaged Models

Large‐scale stone models of maxillary central incisors, canines, premolars and first molars were prepared to represent the selected tooth preparation designs. These models were scanned using a Smart Optics Vinyl Hi‐Resolution 3D Scanner (accuracy: 5 μm), producing greyscale .stl files. These uncoloured digital models were then processed in Meshmixer (v 3.5, Autodesk Inc.), where colour coding was applied to highlight different preparation features. The final 3D IMs were exported as .obj files to create 360° rotatable images, embedded into PDF files and then uploaded to the student learning management system (Canvas) alongside a colour key and guidance for easy access and interaction (Figure 1).

**FIGURE 1:**
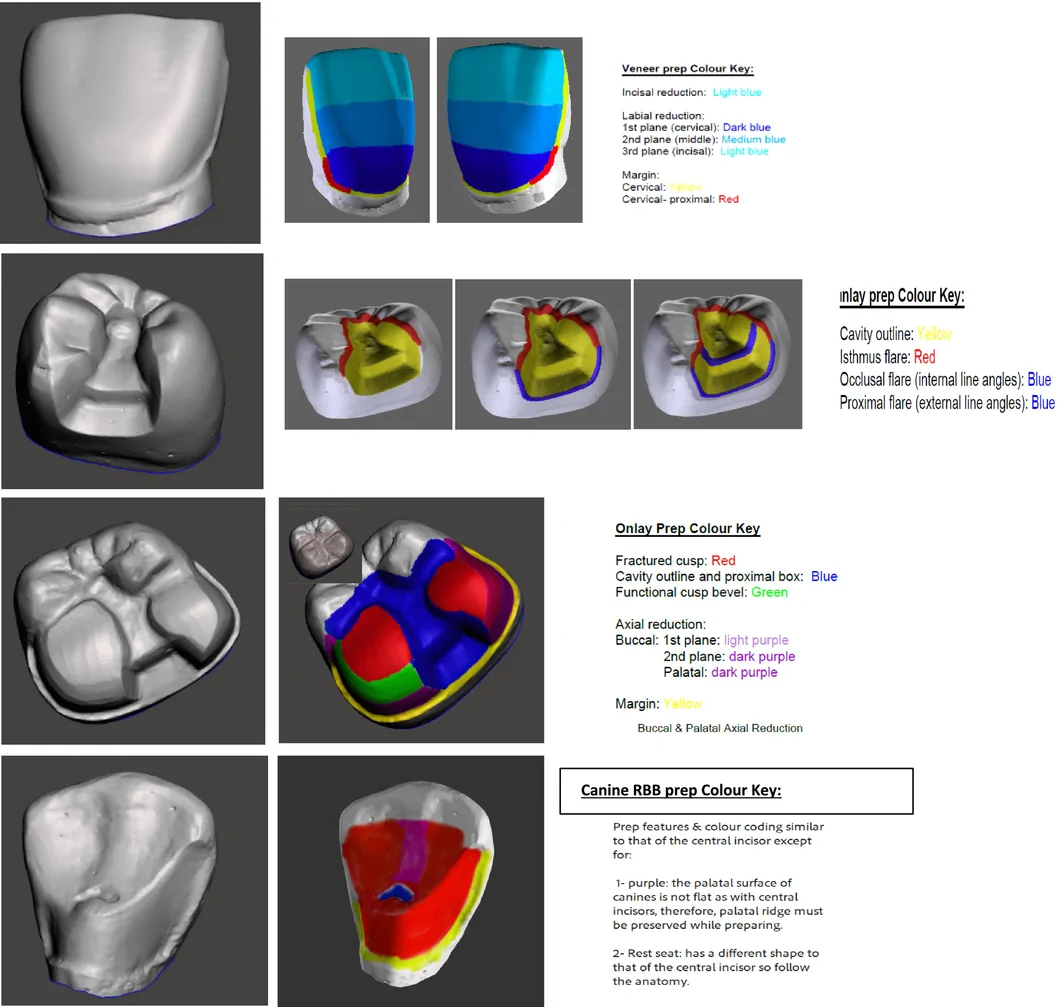
Greyscale 3D imaged models of selected indirect restoration preparations (left) with the corresponding colour‐enhanced models are shown on the right, with the colour keys to facilitate understanding. Shown from top to bottom: central incisor veneer preparation. Staged premolar inlay preparations, illustrating progressive steps from initial cavity outline to final preparation. Molar onlay preparation; an inset image shows the corresponding bonded onlay restoration seated onto the preparation. Canine minimal preparation bridge.

### 2.6 Creating the Physical Reference Models of the Selected Tooth Preparations

All students were provided with a life‐sized physical model of the selected tooth preparations to be used as a physical reference for learning. The physical models represented traditional teaching methods and served as a baseline for comparison with the 3D IMs.

To achieve this, life‐size plastic teeth (Frasaco) were manually prepared to match the selected tooth preparation designs as chosen for the 3D IMs. The prepared teeth were mounted on Frasaco models and then duplicated using an acrylic material to produce mounted study models. This ensured each student had access to a physical model while practising (Figure 2).

**FIGURE 2:**
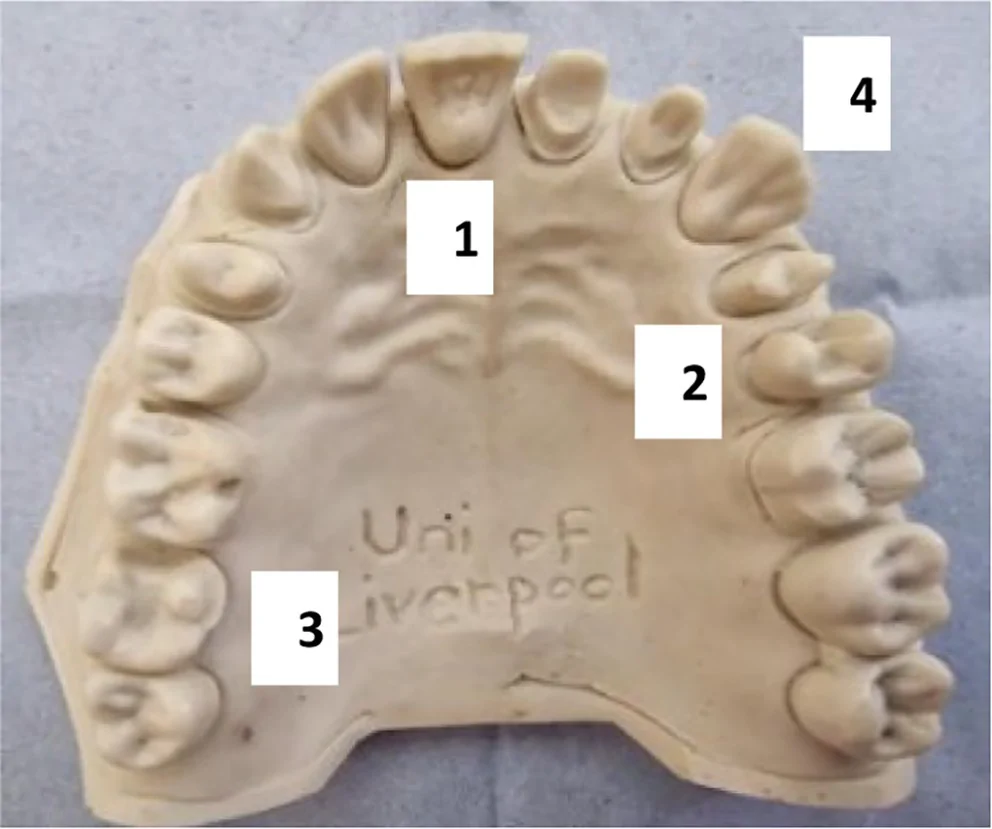
Acrylic physical model featuring the prepared Frasaco teeth used in the study. (1) Central incisor veneer. (2) Premolar inlay. (3) Molar onlay. (4) Canine minimal preparation bridge.

### 2.7 Integration of 3D IMs Into the Course

Students received access to the 3D IMs 1 week before each pre‐clinical session through the school's Canvas platform. A short step‐by‐step guide was provided. During sessions, students could access the 3D IMs on their dental unit screens while practicing the tooth preparations. Physical models were also available for side‐by‐side comparison.

The two cohorts did not experience the same version of the intervention. The 2021 cohort accessed the 3D IMs during their early‐stage rollout under pandemic restrictions, whereas the 2022 cohort used the fully implemented version in a more stable environment. These contextual differences were considered during data analysis and interpretation.

### 2.8 Data Collection

#### 2.8.1 Quantitative Data

A 5‐point Likert scale questionnaire was used to assess students' perceptions of the 3D IMs compared to the physical models. It included questions on the perceived impact of 3D IMs on learning, visualisation and understanding of tooth preparation designs, as well as students' preferences between the two types of models. Open‐ended questions were also included to allow students to provide additional feedback on both resources.

#### 2.8.2 Qualitative Data

Responses to open‐ended questions were analysed using Braun and Clarke's six‐step thematic analysis approach [14]. This included: (1) familiarisation with the data, (2) generating initial codes, (3) searching for themes, (4) reviewing themes, (5) defining and naming themes and (6) producing the final report. Representative quotes were selected to illustrate each key theme.

### 2.9 Questionnaire Design

The questionnaire was designed to assess students' perceptions of the 3D IMs compared to physical models focusing on their impact on learning, visualisation and understanding of TP designs. It consisted of two parts: quantitative Likert‐scale questions and qualitative open‐ended questions.

#### 2.9.1 Quantitative Component

The Likert‐scale questions used a 5‐point scale, with response options ranging from *Strongly Agree (SA)* to *Strongly Disagree (SD)* (Table 1). The questions were designed to be clear, concise and aligned with the study's objectives. Specifically, the questionnaire aimed to:

1. Evaluate students' perceptions of the usefulness *of* 3D IMs in enhancing their understanding of TP designs.
2. Compare students' perceptions of using 3D IMs versus physical models in terms of ease of use, clarity and overall learning experience.
3. Assess students' preferences between the two types of models.

**TABLE 1:** Likert Scale response options and abbreviations.

Response options	Abbreviations
Strongly Agree	SA
Agree	A
Neutral	N
Disagree	D
Strongly Disagree	SD

The Likert‐scale questions included (Table 2):

- Q1: The online 3D IM preparations provided a better visualisation of specific preparation features.
- Q2: The online 3D IMs facilitated the understanding of the tooth preparation designs.
- Q3: Visualising larger‐scale preparations enhanced my self‐assessment skills and helped improve clinical skills.
- Q4: The physical model enhanced the ability to visualize and understand the preparation designs.

**TABLE 2:** Likert Scale statements used in the 3D imaged model questionnaire.

Q1—The online 3D IM preparations provided a better *visualisation* of specific preparation features
Q2—The online 3D IMs facilitated the *understanding* of the teeth preparation designs
Q3—Visualising larger scale preparations enhanced my *self‐assessment* skills and helped improve clinical skills
Q4—The *physical model* enhanced the ability to visualise and understand the preparation designs

#### 2.9.2 Qualitative Component

In addition to the Likert‐scale questions, participants were invited to provide open‐ended questions aimed at gathering deeper insights into their experiences and suggestions for improvement. The following prompts were included:

1. ‘What other preparation designs would you like to see included as online 3D IMs resources?’
2. ‘How do you think the 3D IMs could be enhanced?’
3. ‘What additions or modifications would you suggest for the 3D IMs?’

### 2.10 Data Analysis

#### 2.10.1 Quantitative Analysis

Statistical analysis was conducted using SPSS (Version 28). Descriptive statistics, including means, standard deviations and frequency counts, were used to summarise responses to the Likert‐scale questions. As the data were ordinal and not assumed to be normally distributed, non‐parametric tests were applied. A *p*‐value of ≤ 0.05 was considered statistically significant.

To compare responses between the 2021 and 2022 cohorts (independent groups), the Mann–Whitney *U* test was used. For within‐cohort comparisons of students' perceptions of 3D IMs versus physical models, the Wilcoxon signed‐rank test was applied. Spearman's rank‐order correlation assessed relationships between Likert‐scale items.

To evaluate the internal consistency of the questionnaire, Cronbach's alpha was calculated, yielding a value of 0.585, indicating moderate reliability. Additionally, Cohen's ds was calculated to assess the effect size of cohort differences for each question. Cohen's ds, which accounts for differences in group sizes, was interpreted using standard thresholds: ds = 0.2 (small), ds = 0.5 (medium) and ds = 0.8 (large).

#### 2.10.2 Qualitative Analysis

Open‐ended responses were analysed using Braun and Clarke six‐step approach thematic analysis [15]. This involved familiarisation with the data, generating initial codes, theme identification, theme review, defining and naming themes and final reporting. Representative quotes were selected to illustrate key themes.

#### 2.10.3 Validation and Reliability

The questionnaire was pilot tested with a small group of students (*N* = 10) to ensure clarity and relevance. Feedback from this group was used to refine the wording of the questions. Cronbach's alpha (*α* = 0.585) indicated moderate internal consistency of the Likert‐scale items, which is acceptable given the exploratory nature of the study and the diversity of constructs being measured.

## 3 Results

This section presents the quantitative and qualitative findings of the study in two parts. First, the quantitative results from the Likert‐scale questions, followed by the qualitative themes identified from the open‐ended responses.

### 3.1 Quantitative Results

#### 3.1.1 Descriptive Statistics

On the 5‐point Likert‐scale used in this study, a mean of 3 represents a neutral response. Means above 3 indicate a more positive perception, while means below 3 indicate a less positive perception.

The Likert‐scale responses gave an initial overview of the students' perceptions of the 3D IMs and physical models. The mean scores (± standard deviation) were as follows:

- Q1—visualising preparation designs: Mean = 2.9 (± 0.8). This reflects responses close to the neutral point with a slight positive tendency.
- Q2—understanding tooth preparation designs: Mean = 2.7 (± 0.7). This indicates responses just below the neutral point.
- Q3—enhancing self‐assessment skills: Mean = 3.0 (± 0.6). This reflects responses at the neutral point.
- Q4—preference for physical models: Mean = 2.6 (± 0.8). This reflects responses just below the neutral point.

To further illustrate the distribution of responses, Figures 3 and 4 show the breakdown of Likert‐scale answers by cohort for each question. These highlight differences in perceptions between the 2021 and 2022 groups, particularly in their ratings of 3D IMs for visualisation (Q1), understanding (Q2) and self‐assessment (Q3), as well as their preference for physical models (Q4).

**FIGURE 3:**
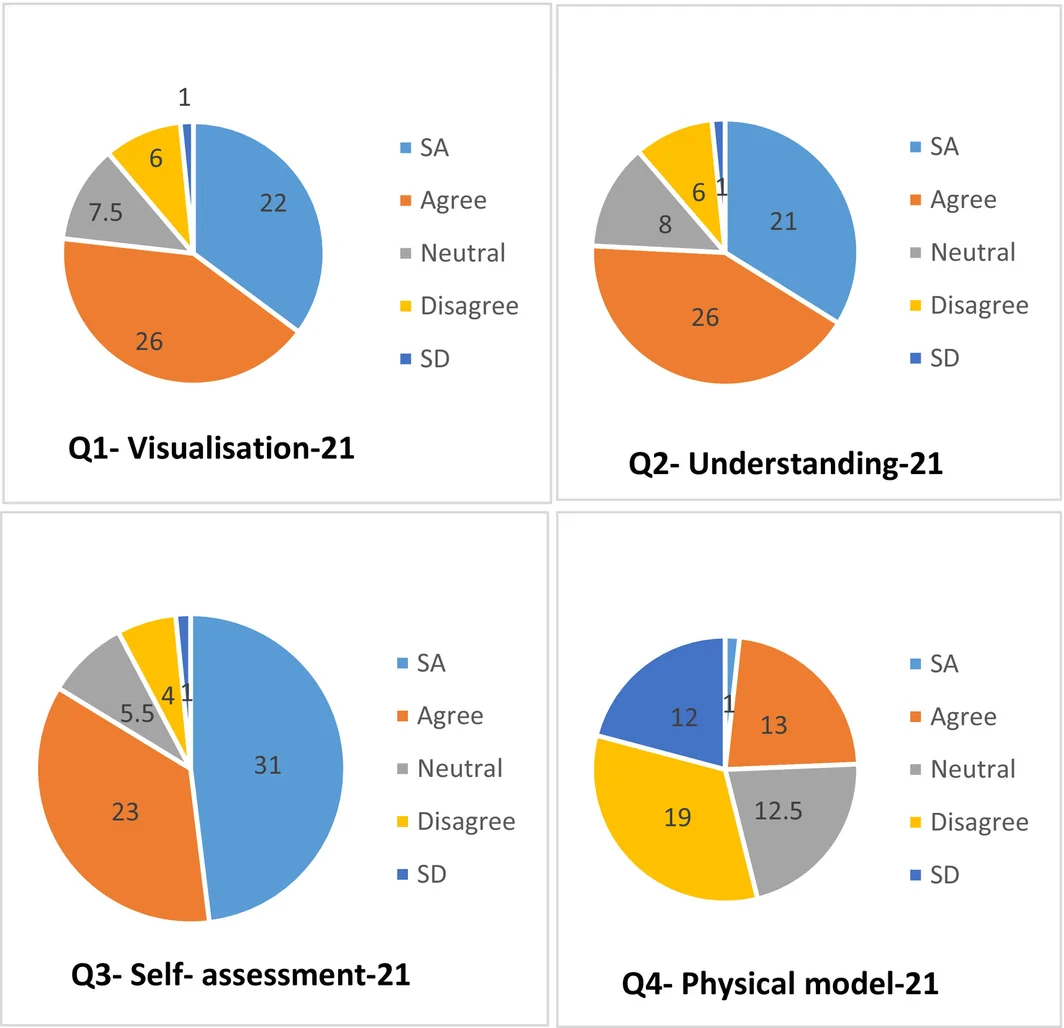
The distribution of individual Likert Scale Responses categories: Strongly Agree = light blue; Agree = brown; Neutral = grey; Disagree = yellow; Strongly Disagree = dark blue for each question in the 2021 cohort.

**FIGURE 4:**
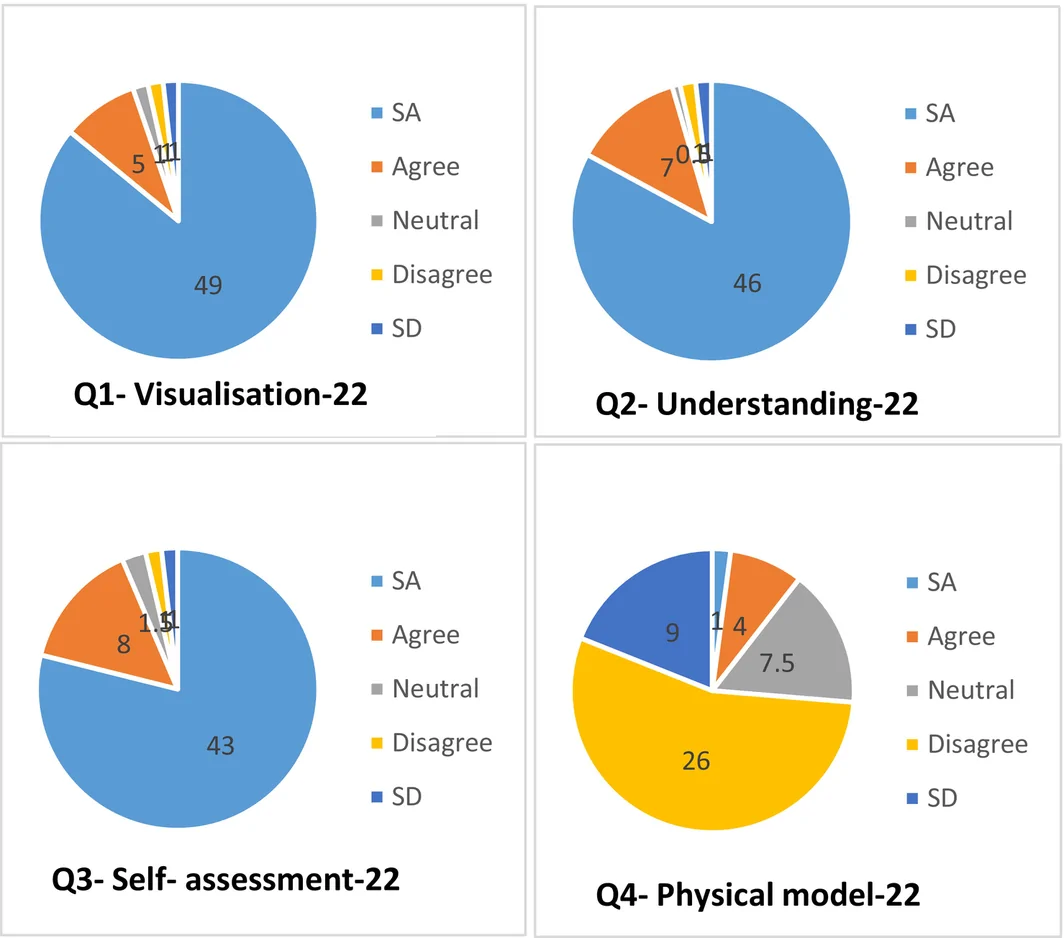
The distribution of individual Likert Scale Responses categories: Strongly Agree = light blue; Agree = brown; Neutral = grey; Disagree = yellow; Strongly Disagree = dark blue for each question in the 2022 cohort.

#### 3.1.2 Comparison Between 2021 and 2022 Cohorts

Because the two cohorts experienced different teaching contexts and versions of the 3D IMs (remote versus face‐to‐face teaching, early rollout versus full implementation), the following between‐ cohort comparisons are presented as exploratory contrasts rather than definitive evidence of the intervention's effectiveness. The Mann–Whitney *U* test showed statistically significant differences between the two cohorts in their perceptions of 3D IMs (Table 3), with the 2022 group consistently rating the 3D IMs more positively:

- Q1—visualising designs: 2022 cohort mean rank = 78.65; 2021 = 40.72 (*p* < 0.001, Cohen's ds = 1.584; very large effect)
- Q2—understanding designs: 2022 = 77.75; 2021 = 41.87 (*p* < 0.001, ds = 1.370; very large effect)
- Q3—self‐assessment skills: 2022 = 71.83; 2021 = 49.44 (*p* < 0.001, ds = 0.775; medium to large effect)

**TABLE 3:** Mann–Whitney *U* test results comparing Likert scale responses between the 2021 and 2022 cohorts for Q1 (visualisation), Q2 (understanding), Q3 (self‐assessment) and Q4 (physical model).

Questions	Group	*N*	Mean rank	Sum of ranks	*p*
Q1	2021	69	40.72	2199.00	< 0.001
Q1	2022	54	78.65	5427.00	
Q2	2021	69	41.87	2261.00	< 0.001
Q2	2022	54	77.75	5365.00	
Q3	2021	69	49.44	2670.00	< 0.001
Q3	2022	54	71.83	4956.00	
Q4	2021	69	68.44	3930.50	0.063
Q4	2022	54	56.96	3695.50	

*Note: N* = number of participants in each cohort. Mean Rank represents relative agreement within each group. Statistical significance set at *p* < 0.05.

There was no significant difference in responses to Q4 (Physical Models) between the cohorts (*p* = 0.063). The effect size (Cohen's *d* = −0.336) suggested a small to medium difference, with the 2022 group showing a slightly greater preference for physical models.

#### 3.1.3 Correlation Analysis

Spearman's rank‐order correlations (Table 4) were performed on the combined responses from both cohorts (*N* = 123). Because the two cohorts experienced different contexts and versions of 3D IMs, these correlations presented as descriptively due to cohort differences. The analysis revealed strong positive relationships between key areas:

- Q1 (Visualisation) and Q2 (Understanding): *r* = 0.891, *p* < 0.001
- Q2 (Understanding) and Q3 (Self‐Assessment): *r* = 0.712, *p* < 0.001
- Q4 (Preference for physical models) showed weak negative correlations with the other items (*r* = −0.164 to −0.240), suggesting that students who preferred physical models tended to find the 3D IMs less beneficial.

**TABLE 4:** Spearman's rank‐order correlations between Likert scale questions (combined data from both cohorts). Q1 (visualisation), Q2 (understanding), Q3 (self‐assessment) and Q4 (physical model).

Question pair	Correlation (*r*)
Q1 and Q2	0.891**
Q1 and Q3	0.689**
Q1 and Q4	−0.164
Q2 and Q3	0.712**
Q2 and Q4	−0.240**
Q3 and Q4	−0.192*

* *p* < 0.05 (2‐tailed).

** *p* < 0.01 (2‐tailed).

### 3.2 Qualitative Results

Thematic analysis [15] of the open‐ended responses revealed key themes reflecting students' perceptions of the 3D IMs and physical models. Themes are presented by cohort (2021 and 2022), with representative quotes used to illustrate the range of student experiences and suggestions.

#### 3.2.1 Thematic Analysis by Cohort

##### 3.2.1.1 2021 Cohort

###### 3.2.1.1.1 Theme 1: Mixed Perceptions of Using 3D IM

Students expressed a range of views about the usefulness of the 3D IMs. While some appreciated the visual support, others preferred hands‐on interaction with physical models or felt the 3D IMs needed further development.

- ‘Maybe if we could move the models around to any angle rather than just front and side.’
- ‘I didn't really bother looking at the 3D IMs, to be honest.’
- ‘I'm a person who has to do preps to learn, and I personally did not find that the 3D image would assist in my learning.’

###### 3.2.1.1.2 Theme 2: Technical Access and Usability Challenges

Several students encountered difficulties accessing the online 3D IMs, especially on specific browsers or devices.

- ‘I struggled to access the resources on my laptop (MacBook using Chrome browser).’
- ‘Hard to access online.’
- ‘Easier to access.’

###### 3.2.1.1.3 Theme 3: Suggestions for Enhancing Physical Models

Some students advocated for expanding the physical model collection to include more preparation types or integrating them with digital resources.

- ‘Physical model of bridge preps.’
- ‘Add minimally‐ prepared bridge preparations on lower teeth to the physical model.’
- ‘If we could have 3D IMs in the simulation suite with prepared teeth to see the dimensions in person or what an ideal prep should look like.’

###### 3.2.1.1.4 Theme 4: Broader Curriculum Applications

Participants proposed future uses of the 3D IMs beyond bridge preparations, particularly for learning basic tooth morphology.

- ‘I liked the 3D models to allow a more detailed look.’
- ‘Could be a useful resource for learning tooth morphology for lower years.’

##### 3.2.1.2 2022 Cohort

###### 3.2.1.2.1 Theme 1: Positive Reception and Expanded Interest

Students generally found the 3D IMs helpful for visualising tooth preparations and suggested expanding the range of available designs.

- ‘I found the current range of models helpful, but it would be great to include lower molar tooth preparations.’
- ‘Any and all preps we have done up until this stage would be helpful.’
- ‘Different types of Onlay preparations and gold shell crowns.’

###### 3.2.1.2.2 Theme 2: Recommendations for Improving 3D IMs

Students recommended adding annotations, measurement indicators and more interactivity to the 3D IMs to improve their educational value.

- ‘Maybe add annotations to the page to give tips on how to angle burs.’
- ‘More annotations of measurements.’
- ‘Have measurements added.’

###### 3.2.1.2.3 Theme 3: Requests for Additional or Varied Physical Models

While appreciating the digital models, students still valued physical models and encouraged their complementary use in the curriculum.

- ‘Physical model of bridge preps.’
- ‘Use a variety of different teeth.’

###### 3.2.1.2.4 Theme 4: Integration Into Earlier Learning

Students expressed that access to 3D IMs in earlier stages of the curriculum would have enhanced their foundational learning.

- ‘Thinking of younger years, it might be useful if they could visualise MOD restorations.’
- ‘These would have been useful when we were learning to do cavity preps in 2nd year.’

##### 3.2.1.3 Summary

Overall, students' perceptions of 3D IMs became more positive in the 2022 cohort, reflecting improvements in access, usability and relevance. While earlier responses highlighted usability issues and preference for physical models, the later cohort showed greater appreciation of the digital resources and offered constructive suggestions for ongoing enhancement and curricular integration.

## 4 Discussion

This study evaluated students' perceptions of 3D IMs compared to physical models in a pre‐clinical dental setting. The quantitative results revealed that students generally found the 3D IMs beneficial, particularly in visualising preparation designs and enhancing self‐assessment. Notably, perceptions improved in the 2022 cohort following refinements in the models and greater curricular integration. Non‐parametric tests were used for the analysis due to the ordinal nature of Likert scale data, which are not assumed to follow a normal distribution. Therefore, data normalisation was not performed, as Likert responses are best analysed using statistical methods appropriate for ordinal data [15, 16].

### 4.1 Key Findings

Students reported that the 3D IMs supported their visualisation of tooth preparations, particularly among the 2022 cohort [6, 7]. However, statistical testing (Mann‐ Whitney) was only applied to compare responses between the two cohorts; no overall significance testing was performed for visualisation scores. These findings therefore represent observed patterns in the data rather than statistically confirmed improvements. This pattern is consistent with broader research on the educational impact of 3D and digital learning resources in health and science education [17, 18, 19]. Their ability to present intricate details of preparation designs allowed for a more comprehensive understanding of both tooth morphology and procedural requirements. This visual depth appeared to support students in recognising fine anatomical and technical details, strengthening their ability to mentally visualise ideal preparations [20].

Descriptive statistics from the questionnaire responses indicated that students found the 3D IMs moderately to highly effective for visualisation, understanding preparation designs and supporting self‐assessment [21, 22, 23, 24]. While the overall preference leaned towards 3D IMs, the tactile feedback offered by physical models remained valuable to some students. Both year groups agreed that the 3D IMs promoted a deeper understanding of the intricacies involved in tooth preparation, contributing to improved confidence and procedural accuracy [25, 26]. Additionally, the ability to compare personal work against ideal digital models promoted self‐reflection and encouraged independent learning: a key component of developing clinical competence [6, 7].

It is worth noting that a minority of students expressed a preference for physical models, potentially due to the hands‐on learning experience they provide. This finding aligns with prior studies suggesting that individual learning styles and sensory preferences can influence resource effectiveness [27].

### 4.2 Variations Between Cohorts

Because the 2021 and 2022 experienced different contexts and versions of the 3D IMs, they were analysed separately wherever possible and any combined or cross‐cohort comparisons are presented descriptively and interpreted as exploratory rather than inferential tests of effectiveness. The observed differences between the 2021 and 2022 cohorts may be attributed in part to the differing educational contexts shaped by the COVID‐19 pandemic. The 2022 cohort, entering their clinical training after the peak of the pandemic, had more consistent access to in‐person instruction and greater familiarity with digital learning tools. For instance, 90% of the 2022 students reported finding the 3D IMs highly beneficial for visualisation, compared to just 50% of the 2021 cohort. Similarly, 80% of the 2022 students indicated that the 3D IMs positively supported their self‐assessment abilities, compared to 55% in the earlier cohort.

In contrast, the 2021 students began their training during a period of abrupt shifts to remote learning, limited face‐to‐face teaching and broader uncertainty. These disruptions likely affected their ability to fully engage with and benefit from digital resources such as the 3D IMs. It is also possible that lower engagement by the 2021 cohort reflected the early stage of the 3D IMs rollout rather than an inherent limitation of the resource itself. At that time, the models had not yet undergone full testing and refinement, and some students reported technical or access issues. These factors, combined with the sudden shift to remote learning, may have limited their ability to benefit fully from the digital resources. This interpretation is supported by studies showing that students learning during the early stages of the pandemic reported greater difficulty adapting to online formats and experienced reduced engagement and satisfaction compared to later cohorts [28, 29]. This may help explain the stronger preference for physical models among 2021 students, with 19% indicating that physical models enhanced their understanding, compared to just 7% of the 2022 students. Because the two cohorts experienced different teaching contexts and versions of the 3D IMs, any combined or averaged results across cohorts should be regarded as descriptive only. Such aggregation may obscure cohort‐specific differences and is not intended as a definitive measure of the intervention's effectiveness. As this analysis combined responses from two cohorts who experienced different contexts and versions of the interventions, the finding should be viewed as exploratory and may have limited generalisability beyond the study setting.

These findings underscore the importance of designing flexible and inclusive dental education strategies that accommodate diverse learning preferences and account for contextual challenges. While 3D IMs offer clear advantages for visualisation and conceptual development, physical models remain essential; especially for learners who benefit from hands‐on, tactile engagement. Future research should explore integrated approaches that maximise the strengths of both modalities.

### 4.3 Recurring Themes

Thematic analysis of the open‐ended responses revealed several consistent themes across cohorts. Students in the 2021 cohort expressed a range of perspectives on the usefulness of 3D IMs. While some appreciated the visual detail these tools offered, others suggested enhancements, such as improved interactivity (e.g., full rotation or zooming capabilities). Although the 3D IMs were designed to be fully rotatable and zoomable, during the initial 2021 rollout these functions, while available, were sometimes sluggish or less intuitive on certain devices. By the 2022 cohort, the models had been migrated to Sketchfab, providing smoother rotation and zoom, which may explain the reduced number of such comments. A portion of this cohort also voiced a continued preference for physical models, emphasising the value of tactile learning experiences. These observations reflect findings from educational psychology literature that advocate for teaching strategies tailored to diverse cognitive and sensory learning styles [30, 31].

In contrast, the 2022 cohort generally responded more positively to the 3D IMs, citing their ease of access and visual clarity. Students in this group suggested further expansion of the model library; for example, the inclusion of lower molar or more complex preparation types. These responses support the view that digital 3D tools can play a significant role in fostering spatial reasoning and deeper conceptual understanding in clinical education [32].

### 4.4 Implications for Dental Education

The findings of this study emphasise the importance of flexibility and inclusivity in curriculum design. The mixed responses from the 2021 cohort suggest that a one‐size‐fits‐all approach may not effectively support all learners. For some, physical models remain an essential component of their learning process. As such, providing both digital and physical learning modalities is critical to accommodating varying student preferences and abilities [33].

However, the 2022 cohort demonstrated greater receptiveness to the 3D IMs highlights their potential as a core component of preclinical dental training. By offering detailed, accessible, and visually rich representations of ideal preparations, 3D IMs can support independent learning, enhance spatial understanding and promote reflective practice. These findings align with existing research demonstrating the pedagogical value of digital 3D resources in improving both cognitive and technical skills in dental education [34].

#### 4.4.1 Actions Taken to Enhance the Educational Experience

Following students' feedback, several actions have already been implemented to improve the teaching and learning experience. The 3D IMs were migrated to Sketchfab, an online platform that enables interactive viewing of 3D models directly within a web browser. Sketchfab was selected for its user‐friendly interface, compatibility across devices and ability to add detailed annotations to different coloured sections of the models to support visual learning (Figure 5). This allowed students to intuitively rotate, zoom and explore the models anytime and anywhere. Interactive annotations were embedded within each model to highlight and explain clinically relevant preparation features and to link visual cues to clinical decision making (Figure 5). In addition, both the physical and 3D IMs libraries were expanded to include a broader range of bridge designs and tooth preparations. This was done to better represent the diversity of clinical cases and to give students more comprehensive exposure to different types of fixed prosthodontic procedures.

**FIGURE 5:**
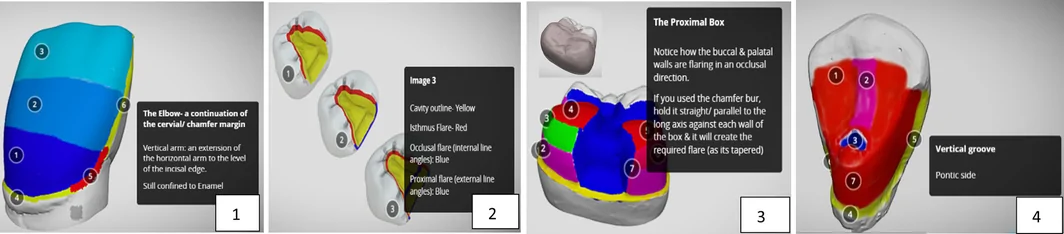
Examples of annotated and colour‐coded 3D imaged models used in simulation teaching. Each numbered marker highlights a specific feature of the preparation; clicking a number on the interactive model displays an explanatory description. From left to right: central incisor veneer preparation showing features such as incisal reduction and elbow margin continuation. Staged premolar inlay preparations, illustrating progressive steps from initial cavity outline to final preparation. Molar onlay preparation with colour‐coded features such as occlusal reductions and margin designs; inset image shows the corresponding bonded onlay restoration seated onto the preparation. Canine minimal preparation bridge with highlighted features including pontic‐side vertical grooves and wing preparation margins.

### 4.5 Limitations

This study provides valuable insights into the integration of 3D IMs in pre‐clinical fixed prosthodontics teaching; however, several limitations should be acknowledged. The modest number of open‐ended responses provided helpful insights but highlighted the need for a larger sample size to gather a broader range of student perspectives. The survey showed moderate internal consistency (Cronbach's Alpha = 0.585), underscoring the importance of refining the survey tool for future studies. Additionally, thematic analysis was conducted by a single investigator, which may introduce subjectivity; emphasising the need for multiple reviewers in future research. Although Cohen's *d* was used to assess differences between cohorts, effect sizes should be interpreted in the context of the exploratory nature of the study. Finally, two of the open‐ended questions were similar in scope and all were positively framed, which may have encouraged constructive suggestions over criticisms and limited the expression of negative views. These limitations should be considered when interpreting the findings.

### 4.6 Future Plans

To address these limitations and further enhance students' learning experience, the following steps are planned. The survey tool will be reviewed and piloted to improve internal consistency and ensure alignment with the constructs being measured, thereby enhancing the reliability of future surveys. Qualitative data collection will be expanded to include a broader range of student perspectives, enriching the analysis and future thematic analysis will involve multiple examiners to reduce bias and increase credibility. Effect sizes will be more clearly contextualised in future studies to emphasise their exploratory nature and the practical significance of the findings in dental education, including fixed prosthodontics. Finally, the open‐ended questions will be redesigned to include neutral prompts as well as opportunities to share criticism, reducing positive framing bias and ensuring that both favourable and unfavourable student perceptions are recorded more fully.

## 5 Conclusion

This study evaluated the students' perception of 3D IMs alongside physical models in pre‐clinical fixed prosthodontics. The findings show that, when fully implemented and supported within the curriculum, 3D IMs can assist students in visualising tooth preparation designs, understanding key features and developing self‐assessment skills, while physical models continue to provide valuable tactile learning experiences. Differences between the 2021 and 2022 cohorts reflect both pandemic‐related disruption and the stages rollout on the 3D IMs; therefore, the results should be interpreted cautiously and not as evidence of a definitive effect.

Taken together, the study highlights the value of providing students with complementary digital and physical learning resources tailored to diverse preferences and learning contexts. Future work should assess the long‐term impact of 3D IMs on knowledge retention, confidence, and clinical performance and explore optimal ways to integrate these tools across different stages of dental education.

By combining innovative resources with student feedback, dental schools can build more inclusive, flexible and effective pre‐clinical training environments that better prepare learners for the complexity of dental practice.

## Funding

The authors have nothing to report.

## Conflicts of Interest

The authors declare no conflicts of interest.

## Data Availability

The authors have nothing to report.
